# Supplementary figures and images for: Mitochondrial dysfunction induces RNA interference in C. elegans through a pathway homologous to the mammalian RIG-I antiviral response
Source: PLoS Biol. 2020 Dec 2;18(12):e3000996. doi: 10.1371/journal.pbio.3000996 (PMC7735679; doi:10.1371/journal.pbio.3000996)

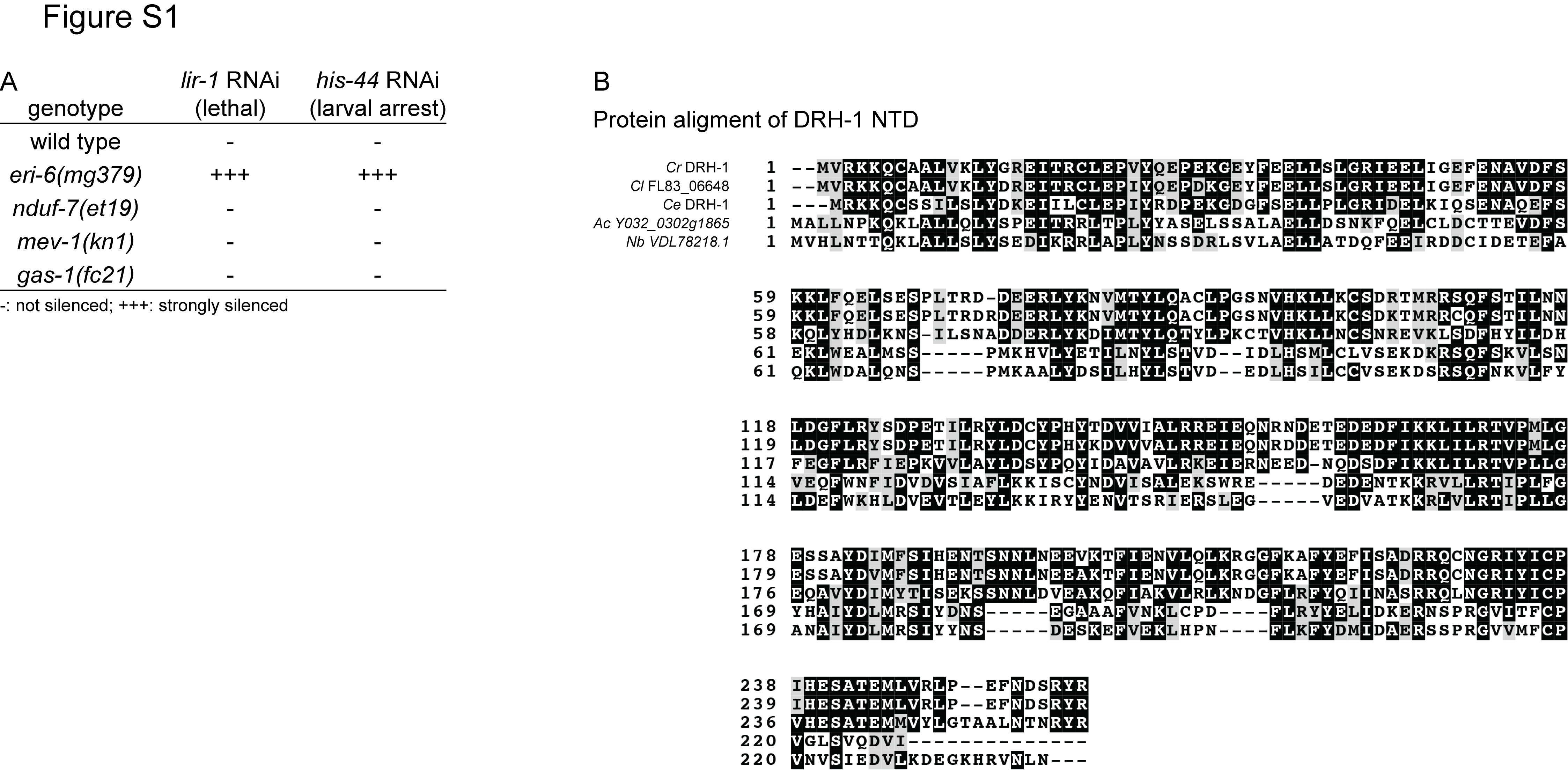

Supplement: S1 Fig — (A) Enhanced RNAi response to lir-1 RNAi or his-44 RNAi in the eri-6 control enhanced RNAi mutant or any of the mitochondrial mutants (nduf-7, mev-1, or gas-1), causes lethality/arrest on the mitochondrial mutants but not wild type. (B) Protein alignment of DRH-1 NTD in nematode species. Ac: Ancylostoma ceylanicum; Ce: Caenorhabditis elegans; Cl: Caenorhabditis latens; Cr: Caenorhabditis remanei; Nb: Nippostrongylus brasiliensis; NTD, N-terminal domain; RNAi, RNA interference. (TIF) [file pbio.3000996.s001.tif]

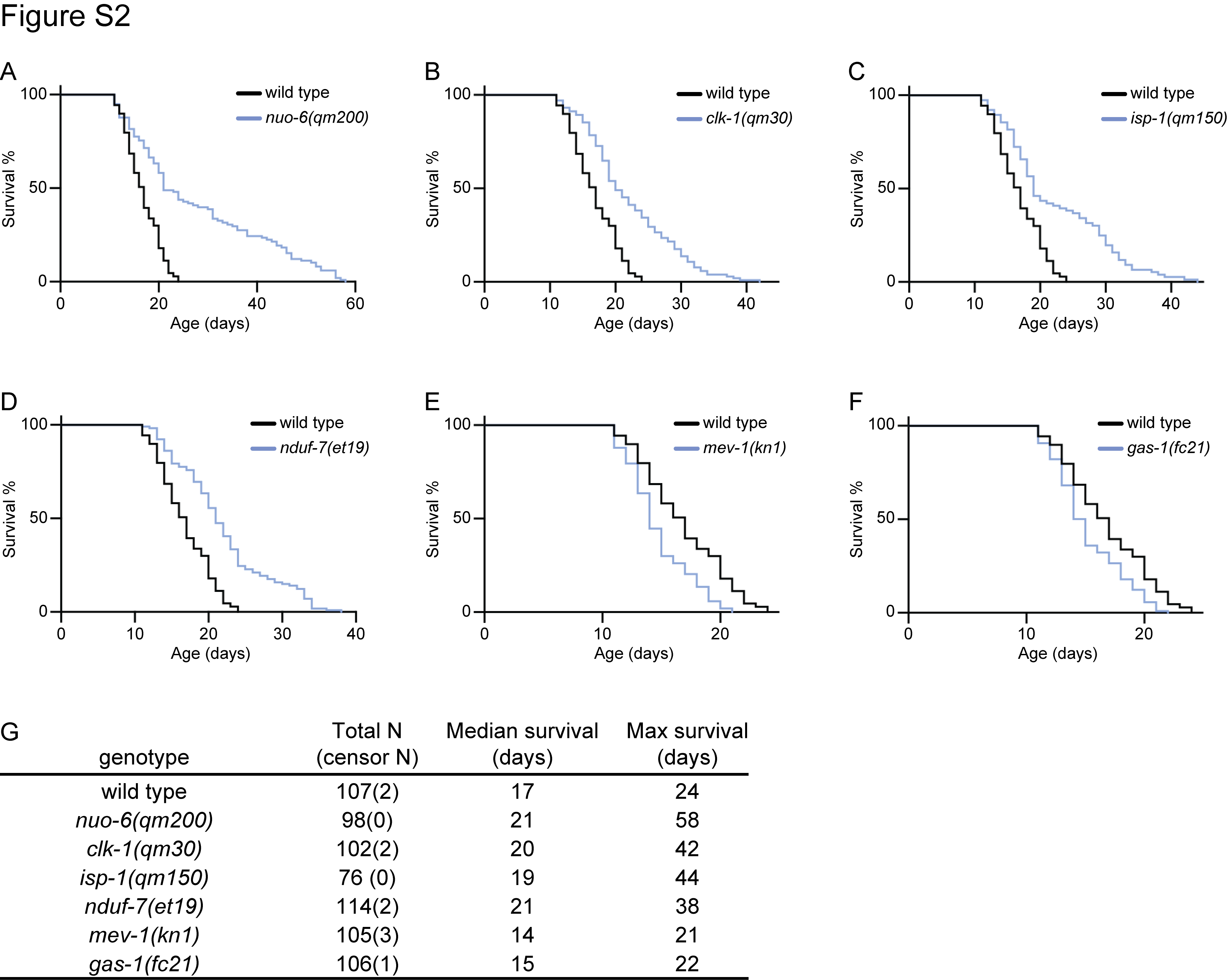

Supplement: S2 Fig — (A–D) Mitochondrial mutants (nuo-6, clk-1, isp-1, or nduf-7) extend life span. (E and F) Mitochondrial mutants (mev-1 or gas-1) shorten life span. (G) Median and maximal survival and number of animals surveyed and censored due to losses during the month-long assay. The underlying numerical data can be found in S1 data. (TIF) [file pbio.3000996.s002.tif]

ATP5A

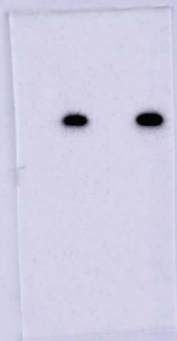

Actin

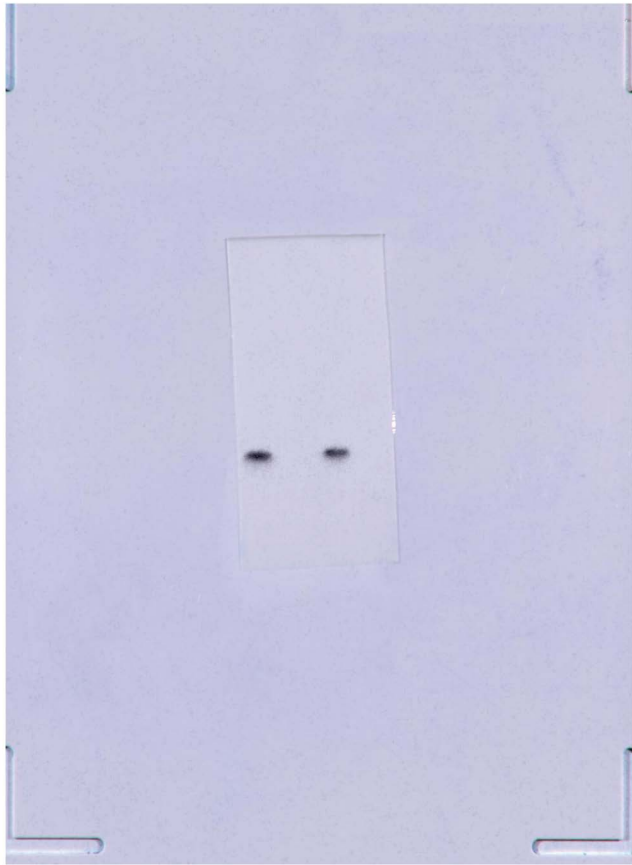

Supplement: S1 Raw images — (PDF) [file pbio.3000996.s007.pdf]
