# Supplementary material for: Mitochondrial dysfunction induces RNA interference in C. elegans through a pathway homologous to the mammalian RIG-I antiviral response
Source: PLoS Biol. 2020 Dec 2;18(12):e3000996. doi: 10.1371/journal.pbio.3000996 (PMC7735679; doi:10.1371/journal.pbio.3000996)
Supplement: Attachment — Submitted filename: Response to Plos Biol 10-2.docx [file pbio.3000996.s008.docx]

Response to reviewers Oct 2, 2020:

Ines Alvarez-Garcia, PhD,
Senior Editor,
[ialvarez-garcia@plos.org](mailto:ialvarez-garcia@plos.org),
PLOS Biology

Dear Dr. Garcia

Below are our responses, **in bold blue**, to the generally supportive comments *(in italics)* on our manuscript.

***Rev. 1: Oded Rechavi –***  *This manuscript was a pleasure to read and I think it would be of interest to many readers from different fields, and especially to researchers studying RNAi. Briefly (I won't summarize all the discoveries that they made), they show that worms sense the state of their mitochondria, and that upon disruption of mitochondrial functions they mount an antiviral RNAi response. They follow the mechanism and find that how it involves eol-1, drh-1, and potentially secretion of dsRNA to the cytosol. I like the paper as is but* ***here are some small suggestions****:

Introduction:
- The introduction might benefit from more references. For example, the entire part about RdRP is unreferenced. This could be important because, for instance, the paragraph that contains these sentences otherwise sounds very speculative: "RNA viruses of course depend on such RdRp genes for their replication so the defense pathway may have evolved from the viral weapon, or vice versa. The fact that RdRp genes mediate RNAi functions across eukaryotic phylogeny suggests that these pathways have been lost for example in most of the vertebrate lineages that only have the first stage of RNAi, the Argonautes and Dicer, rather than RdRps evolving or being acquired by horizontal transfer from viruses independently in so many eukaryotic lineage" - There's literature about this, for example from Eugene Koonin's lab. I am not an RdRP expert but as far as I know viral RdRPs and the RdRPs of animals evolved independently (I was under the impression that unlike viral RdRPs, the RdRPs of
animals evolved from DNA dependent RNA polymerases).*

**Yes, our presentation of RdRp phylogeny is not the standard presentation of it, which generally stresses the ubiquity of the second stage of RNAi in fungi and plants and a few animals. But this eukaryotic distribution strongly favors that the ancestral eukaryote carried the RdRp RNAi supercharging and that most animals have displaced it with something else, most likely an amped up innate immune system and B cells and T cells in vertebrates. We are in the process of writing up a manuscript that deeply explores the phylogenetic distribution of RdRp genes, but at present it is not intimately connected to the coupling of mitochondrial dysfunction to the enhancement of RNAi that we document in this paper. Because it does not intersect (at present) with the mitochondrial RNAi axis of this paper, we have deleted the discussion of RdRp phylogeny in the introduction where it is superfluous but have a short section in the Discussion because it is so relevant to why worms have some decorations to their RNAi pathway. And we have added this reference to the origin of eukaryotic RdRP genes** (Iyer et al., 2003)**.**

*Results:
- Is it possible to track the mitochondrial RNA using smFISH to see if it's released to the cytosol? I think this part is less convincing, but the authors don't write this part carefully, so it's ok (They only say that the process "might" involved secretion of dsRNA). To show directly an increase (using smFISH) in cytosolic RNA would help substantiate the claim.*

**It is possible that smFISH could detect mitochondrial RNA in the cytosol, but it is not as simple as just counting dots of mitochondrial genomic RNA in the cytoplasm. First, any dots from smFISH hybridization are difficult to assign to cytoplasmic localization or even to individual cells. Are the dots within mitochondria or outside of mitochondria? So there must be high resolution co-staining. And as the reviewer mentions, we propose that dsRNA is released from dysfunctional mitochondria to activate enhanced RNAi. We would need to do smFISH in a variety of enhanced RNAi mutants, for example, in the *eri-1, eri-6/7,* synMuvB, and *rrf-3* class and compare to the *hsp-6* or *nduf-7* mitochondrial mutants. We do not envision this as a simple one-off experiment but as a year of work that would give a rather unsatisfying mapping of dots all over the *C. elegans* body. And during mitochondrial dysfunction, there is likely to be mitophagy, a lysosomal event that will not be revealed by the rough mapping of smFISH dots to the approximate locations of *C. elegans* cells or the cytoplasm of *C. elegans* cells. And this is an entirely different set of tools than we are equipped to study, especially during a Covid pandemic.**

**But we have added computational analysis to address the possible production of siRNAs from mitochondrial transcripts. We now include deep siRNA sequencing data from wild type and enhanced RNAi mutants that show that mitochondrially translated mRNAs generate very abundant siRNAs in both wild type and enhanced RNAi mutant strains (Supplemental Table 2). For example, the mitochondrial mRNAs encoding *ctc-1*, *nduo-2*, and *nduo-5*, mitochondrial electron transport chain genes, generate some of the most abundant siRNAs of any genes, at levels near those siRNAs generated by transposable elements in *C. elegans* (the *retr-1* transposable element at rank 24, *ctc-1* at rank 29, *nduo-*2 is at rank 104, *nduo-5* at rank 119). No nuclearly-encoded mitochondrial genes are in this top rank of siRNA producing loci. This is now incorporated in the paper.**

*- Of course one could dig further to study how drh-1 and eol-1 mediate the lifespan extension of the mitochondrial mutants, but I think this is beyond the scope of the paper. This part, as is, feels a little out of tune with the rest of the paper, because this is a major discovery (the link to aging) which is not explored (again because it's out of scope which is ok). Perhaps this could be improved by moving this to the discussion or expanding on the theory. If I understand correctly the proposed mechanism is a tradeoff of increased longevity at the price of vulnerability to viruses at old age?*

**We have clarified the logic of the analysis but because it presents interesting lifespan data, we have not moved this to the Discussion. We think that it is one of the four main take home lessons of our paper: that the enhancement of RNAi by a mitochondrial mutation is a major axis of its lifespan enhancement, that the mitochondrial defect triggers strong antiviral defense, perhaps because of the involvement of mitochondrial functions such as *dhr-1* and *eol-1*, and that the strong antiviral defense is WHY the animals live longer. Given the terrible vulnerability of older humans to Covid19, this is a timely and thought-provoking result that we discuss in the Discussion.**

*- The figures in the PDF that I downloaded from the PLoS Bio site are in very low resolution (probably something about the upload to the site) that makes it hard to assess certain observations (most notably in the microscopy pictures, e.g. aggregation of eol-1).*

**We apologize for this. Somehow in the uploading we lost resolution that our edited manuscript had. This will be corrected in our resubmission. And, with the wonderful non-anonymity of the reviewers, we can simply send you the PDF----what a concept! Even simpler, in the next review, Professors Rechavi and Parker should be able to download the original picture from the journal web site (unfortunately in this Web era, it takes more and more clicks to review papers).**

*discussion:
the discussion could be more organised, perhaps it would help to break it into subsections.
I think that it's worth expanding on the link between this paper and the previous papers from the Ruvkon lab about danger signals in C.elegans (e.g. Melo and Ruvkun, Cell 2012). This paper is referenced but i think it could be discussed further, because conceptually it's highly related: worms sense their internal state (in this case mitochondrial functions) and RNAi is part of the surveillance mechanism.*

**Done.**

*Small typo: the abbreviation RdRP is written in different ways across the paper (RdRp, Rdrp).*
**Done.**


***Rev. 2: Roy Parker***
*In this article, Mao and colleagues describe a process whereby mitochondrial dysfunction [hsp-6(mg585) allele] in nematodes enhances RNAi and triggers antiviral gene induction. The authors show that this process is dependent on DRH1 (a homolog of the mammalian antiviral RIG-I/MDA-5 proteins) and EOL1 (a paralog of the mammalian antiviral Dxo protein). The authors show that hsp-6(mg585) results in increased cytoplasmic levels of mitochondrial RNAs, which is known to trigger the MDA-5/RIG-I-mediated interferon response in mammalian cells. Moreover, the synthesis of mtRNA is required for the formation of ELO-1-containing cytoplasmic foci in response to hsp-6(mg585)-mediated mitochondrial dysfunction. Lastly, the authors show that the hsp-6(mg585) mitochondrial mutant increases longevity, and this increase is dependent on both DRH-1 (RIG-I/MDA-5) and EOL-1 (Dxo). The authors thus propose that the DRH-1/ELO-1 enhanced antiviral defense pathway provides an anti-aging mechanism in response to mitochondrial mutations.

The finding that hsp-6(mg585) is stimulating a RIG-I/MDA-5-like innate antiviral response in C. elegans is an interesting finding since C. elegans lack the MAVS-interferon antiviral response. Thus, this finding provides insight into previous, orthologous and/or alternative functions of mammalian interferon response proteins mediated by RIG-I and MDA-5. These finding also show that mitochondrial dysfunction is a conserved mechanism between mammal and C. elegans by which antiviral proteins sense viral infection. Lastly, the finding that EOL-1 is required for this function and forms cytoplasmic foci is interesting, although the significance of these foci remains unclear.
This review is from Roy Parker and I would be willing to clarify these comments for the authors if needed.

Major Comments:
1) As it stands, there is a correlation that some mutations increase mitochondrial dysfunctions, increase RNAi, and increase mitochondrial RNA in the cytosol. The significance of this correlation would be strengthened if they demonstrated that the mitochondrial mutants that do not increase RNAi, do not increase the presence of mitochondrial RNA in cytosol.*

## About half of the 7 mitochondrial mutants that exist strongly enhanced RNAi. *hsp-6, nuo-6, clk-1,* and *isp-1* strongly enhanced RNAi. Three other mitochondrial point mutants, *nduf-7(et19), mev-1(kn1)* and *gas-1(fc21),* did not enhance RNAi. We added these data in a new supplemental Figure 1A. NUO-6, GAS-1, and NDUF-7 are components of complex I, MEV-1 is from complex II, ISP-1 is a component of complex III, and CLK-1 produces the ubiquinone that also functions in electron transport between complex III and I or II. Roy wants us to go more deeply into why some mitochondrial mutations enhance RNAi and why some do not, where instead we explored whether the enhanced RNAi was more like *eri-1* or *rrf-3*, or synMuv, or other enhanced RNAi mutants. To address this question, we have added more analysis of mitochondrial hits from our full genome RNAi screens for enhanced RNAi (Fischer et al, 2013) as well as screens for defective RNAi (Kim et al, 2005). Nuclearly-encoded mitochondrial genes constituted a large class of gene inactivations that cause an RNAi defective phenotype or a transposon activation phenotype (often correlated with RNAi defective): *coq-1*, a coenzyme Q biosynthetic gene (from the Kim et al 2005 full genome screen), *aco-2*, aconitase, *idha-1*, mitochondrial isocitrate dehydrogenase, *asb-1* and *asg-1*, subunits of the mitochondrial F0 ATP synthase, *ucr-2.3*, ubiquinol cytochrome c reductase, *tufm-1*, mitochondrial translation elongation factor, *tin-44* mitochondrial inner membrane translocase, and *phb-2*, prohibitin, a complex that acts as chaperone for electron transport chain components emerged from a collection of 27 gene inactivations that activate transposons in the germline (transposons are silenced by RNAi in the germline (Vastenhouw et al., 2006). Our full genome screen for enhanced RNAi gene inactivations discovered 60 gene inactivations that enhance RNAi, including a number of the genes that we had previously discovered (many of the synMuv B mutations that mediate DNA amplifications, perhaps of various Argonaute and RdRp genes that biochemically present siRNAs to mRNA targets and amplify those siRNAs) as well as the *eri-6/7* and other eri genes discovered in that paper, for example *eri-12* (Fischer et al, 2013). *sdha-2* which encodes a subunit of the mitochondrial succinate dehydrogenase complex (Complex II) also emerged from this Eri screen.

## We don’t yet understand why some mitochondrial deficits cause a defect in RNAi and others cause an enhancement of RNAi. From our decades long study of RNAi pathways, we have discovered that recently acquired viral genome segments are surveilled by a dedicated subset of the RNAi pathway; when those viral genes are not silenced because of a defect in the ERGO-1 Argonaute protein for example, the ensuing viremia (which we documented in the Fischer and Ruvkun PNAS 2020 paper) causes a massive up-regulation of the other RNAi pathway genes, for example other Argonautes and RNA helicases. So mitochondrial players in the viral silencing pathway will cause an Eri phenotype where when they interact with the ERGO-1, ERI-1, or synMuvB pathways, for example. There is precedent for mitochondrial engagement of small RNA pathways, most especially for the piRNAs of Drosophila which depend on the zucchini mitochondrial phosphatase. Interestingly, while the piRNAs are the key transposon silencing pathway in insects, like most animals, Drosophila has jettisoned its RdRp genes and the utility of this second stage siRNA amplification system for viral defense. There is also the complexity that many of the mitochondrial mutations we have analyzed are non-null alleles because many of the genes are essential. These are the rare viable mitochondrial point mutations that the *C. elegans* field has used for a variety of aging and other studies over the years; in most cases inactivation of these nuclear genes that encode mitochondrially localized proteins causes larval arrest. Thus, when one does genetic or RNAi screens, mitochondrial mutations or gene inactivations that emerge tend to be in auxillary or duplicated factors that are not essential. In some screens RNAi after the L4 stage has been used to assess lifespan enhancement or RNAi phenotypes (a so-called P0 RNAi screen of the first generation after feeding commences, compared to an F1 screen of progeny the next generation after dsRNA feeding is commenced). We saw a similar, somewhat mysterious (at present) interaction between some mitochondrial mutant alleles and *E. coli* mutants that fail to remove iron from a siderophore (Govindan et al 2016 PNAS Ros paper). Some ETC mutants were synthetic lethal at the L1 stage (a very severe and obvious phenotype) with *E. coli* siderophore mutants and other ETC mutants were not sick at all.

**So our analysis of the mitochondrial mutants that enhance RNAi and the ones that do not, is unlikely to become easily understandable in a two month revision period and during a Covid attenuated experimentation period. So we decided to not pursue this mystery in this paper. We believe that our genetic pathway analysis of the genes needed for the enhanced RNAi response (e.g. EOL-1, and DRH-1) solved a more important mystery. Why some mitochondrial mutants trigger this response and some do not will be the next mystery we will address.**

*2) As it stands, there is a correlation that some mutations increase mitochondrial dysfunction, RNAi, and lifespan. To show this correlation is pertinent to the increased lifespan, the authors should show that mitochondrial dysfunction that does not increase RNAi does not increase lifespan.*

**We agree that our interpretation is that the mutations that decouple enhanced RNAi from the mitochondrial mutations and also decouple lifespan extension may end up also decoupling mitochondrial mutants that do not enhance RNAi from their lifespan enhancement pathways. We added the lifespan analyses of other mitochondrial mutants, including *nuo-6(qm200)*, *clk-1(qm30)*, *isp-1(qm150)*, *nduf-7(et19)*, *mev-1(kn1)* and *gas-1(fc21)* in a new supplemental Figure 2. Interestingly, mitochondrial mutants that enhance RNAi, such as *hsp-6(mg585)*, *nuo-6(qm200)*, *clk-1(qm30)* and *isp-1(qm150)*, extend lifespan, while mutants that do not enhance RNAi, such as *mev-1(kn1)* and *gas-1(fc21)*, shorten lifespan. The only exception is *nduf-7(et19)*, which does not enhance but extends lifespan.**
 *3) Since there is a substantial literature on how mitochondrial dysfunction increases lifespan (e.g. Zhang et al., 2018, Cell), the work should be put in the context of earlier studies in this area.*

**We have added a discussion of the Zhang and Dillin Wnt signaling pathway to our Discussion. But we want to stress that the Zhang paper explores the signaling pathways that couple mitochondrial stress in neurons to activation of the *hsp-6* mitochondrial response in the intestine. Our paper does not attempt to address any mitokine type of endocrine signaling (and parenthetically, the endocrine responses to tissue-specific mitochondrial dysfunction are quite weak and difficult to study). Such tissue-specific mitochondrial dysfunction experiments are also subject to how perfect any promoter fusion is: if the mitochondrial disruption is driven in one cell type with a cell type-specific promoter, if that promoter is slightly leaky in other tissues, a slight response can be from a leaky expression in the unintended tissue. Because the response in the intestine is a weak response, the chances of weak expression of the mitochondrial disruption protein in the intestine, for example, is a serious possibility. In addition, if mitochondrial stress activates RNAi, as we document in our paper, then transgene reporters may become silenced in both the tissues where there is mitochondrial stress, and given that siRNAs have evolved to spread between cells, the siRNAs themselves may be the mitokine. So one must worry a lot about transgene reporter expression responding to mitochondrial stress. We addressed this explicitly in our paper using qPCR to quantitate endogenous gene expression rather than just depending on transgenes.**

*Additional comments:
4) The title, abstract, and discussion state DRH-1 activity as homologous to RIG-I, but in the results section DRH-1 is primarily stated as an MDA-5 homolog. The authors should provide clarity on this discrepancy since RIG-I and MDA-5 have different RNA substrate preferences in mammalian cells. Based on homology, is DRH-1 expected to have substrate preferences more closely aligned to MDA-5 or RIG-I substrate preferences?*

**In a TreeFam analysis of DRH-1, it is an ortholog of *C. elegans* DRH-3 (named Dicer-Related Helicase for the similarity of the RNA helicase domain of Dicer, the dedicated small RNA nuclease and helicase) and more distantly related but still orthologous to human DDX58 (DExD/H-box helicase 58), also known as RIG-I, to DHX58 (DExH-box helicase 58) also known as RIG-I-like receptor, and to IFIH1 (interferon induced with helicase C domain 1) also known as MDA5 or RIG-I-like receptor 2. Paralogous to these orthologues are the Dicer helicases. So it is not possible to assign DRH-1 to either RIG-I homology or MDA-5 orthology via phylogenetic tree analysis---mammals have simply duplicated this gene family to 3 members and *C. elegans* to 2 members. We agree that in our study, DRH-1 functions like MDA-5, because MDA-5 is the RNA sensor in human that responds to the release of mitochondrial-generated mRNAs to the cytosol. But the substrate preference of DRH-1 is not well studied in *C. elegans*. One possibility is that in evolution, vertebrates developed multiple viral RNA sensors, including RIG-I and MDA-5; however, in *C. elegans* only DRH-1 functions as a viral sensor, since *drh-2* is a pseudogene and *drh-3* encodes a component of an RdRP protein complex. MDA-5 and other paralogs are also named RIG-I-like family members in human, and in the *C. elegans* literature DRH-1 has been RIG-I homology has been noted. Finally, by simple BlastP analysis the probability of the DRH-3, RIG-I homology is e-69, RIG-1 like 3 or MAVS is e-60. So one cannot assign any one to one orthology to a duplicated and diverged gene family. They are ALL beautiful. Thus, we prefer to use DRH-1/RIG-I to simplify the naming and functional description of DRH-1 in *C. elegans*.**

**Below is the Blastp summary for a query DRH-1 to human proteins.**

| RIG-I 251 bits(640) | 7e-69 |  | 218/735(30%) identity |
| --- | --- | --- | --- |

| RIG-1 like 3 227 bits(579) | 1e-60 |  | 214/738(29%) |
| --- | --- | --- | --- |

| MAVS 226 bits(577) | 2e-60 |  | 214/741(29%) | 3 |
| --- | --- | --- | --- | --- |

**See**

**http://www.treefam.org/family/TF330258#tabview=tab1**

*5) References to Figure 5 preceded references to Figure 4 in the text. Moreover, Figure 4 references break up references to Figure 5 sub figures. This is confusing. The authors should consider re-arranging Figures/text to make this section easier to navigate for readers.*

**We apologize that our figure configuration was confusing. The reason we placed some microscopy images into Figure 4, where they more naturally fit in Figure 5 based on order of exposition in the text was to present all of our fluorescent microscopy in Figure 4 with a black background. This is due Gary Ruvkun’s obsession about placing fluorescent images on a black background for ease of viewing. In group meetings, he constantly blurts out to USE A BLACK BACKGROUND so that your audience is not blinded by the white background in a figure also presenting non-fluorescent graphs for example. So we thought that while it is slightly confusing to refer to a fluorescent panel in Figure 4 and then back to Figure 5, it is actually better for the reader to actually see the data, which of course is the most important aspect of writing a paper. But based on this critique, we have re-arranged our figures as followings: the original Figure 5 A&B became new Figure 4 A&B; the original Figure 4 became new Figure 5; the original Figure 5 C&D became new Figure 6 A&B; the original Figure 6 became Figure 7.**

*6) I found the difficult to read, primarily due to too much background information throughout the article, and complicated paragraph structure. This could be improved.*
**We improved the writing.**
